# Electroacupuncture Prevents Osteoarthritis of High-Fat Diet-Induced Obese Rats

**DOI:** 10.1155/2020/9380965

**Published:** 2020-07-06

**Authors:** Lin-Lin Xie, Yu-Li Zhao, Jian Yang, Hui Cheng, Zhen-Dong Zhong, Yi-Ru Liu, Xian-Lun Pang

**Affiliations:** ^1^Diagnosis and Treatment Center of Classical Chinese Medicine, The Affiliated Hospital (TCM) of Southwest Medical University, Luzhou, 646000 Sichuan, China; ^2^Department of Geriatrics, The Affiliated Hospital (TCM) of Southwest Medical University, Luzhou, 646000 Sichuan, China; ^3^Department of Orthopedic Surgery, Yongchuan Hospital of Chongqing Medical University, Chongqing 402160, China; ^4^Clinical Laboratory, Sichuan Academy of Medical Sciences & Sichuan Provincial People's Hospital, Chengdu 610000, China; ^5^Health Management Center, The Affiliated Hospital (TCM) of Southwest Medical University, Luzhou, 646000 Sichuan, China

## Abstract

The effects of acupuncture on osteoarthritis (OA) pathogenesis have been demonstrated in vitro and in animal models. However, the potential for acupuncture to mediate protective effects on obese-induced OA has not been examined. Here, we investigated the effects of different acupuncture patterns on OA pathogenesis in high-fat diet- (HFD-) induced obese rats. After 12-week diet-induced obesity, obese rats were treated with three acupuncture protocols for 2 weeks, including ST36, GB34, and ST36+GB34. The results showed that the three acupuncture protocols both prevented obesity-induced cartilage matrix degradation and MMP expression and mitigated obesity-induced systemic and local inflammation but had different regulatory effects on lipid metabolism and gut microbiota disorder of obese-induced OA rats. Furthermore, the three acupuncture protocols increased the microbial diversity and altered the structure of community of feces in obese rats. We found that ST36 and GB34 could inhibit proinflammatory shift in the gut microbiome with an increase in the ratio of Bacteroidetes/Firmicutes and promote the recovery of relative abundance of *Clostridium*, *Akkermansia*, *Butyricimonas*, and *Lactococcus*. Although both ST36 and GB34 had an anti-inflammatory effect on serum inflammatory mediators, only the acupuncture protocol with both ST36 and GB34 could effectively inhibit LPS-mediated joint inflammation in obesity rats. Therefore, relieving obesity-related chronic inflammation, lipid metabolism disorder, and gut microbiota disorder may be an important mechanism for acupuncture with ST36 and GB34 to promote OA recovery.

## 1. Introduction

Osteoarthritis (OA) is a common chronic disorder of the joints, characterized by progressive loss of articular cartilage, osteophytes, and synovitis. In OA pathogenesis, obesity is regarded as a risk factor for the onset and increased rate of progression of metabolic OA in joints [1]. Historically, excessive weight increases the mechanical loads on the knee joint to result in wear and tear on joints, which is the most likely mechanism through which obesity contributes to OA [2]. However, it has been shown that obesity is also a risk factor for OA in non-weight-bearing joints, like hand and wrist [3], which indicates that excessive joint load cannot fully explain the link between OA and obesity.

Emerging evidence suggests that adipose tissue inflammation and disturbed lipid metabolism may be sufficient to lead to the onset and progression of OA [4–6]. There is abundant research data suggesting that intrinsic inflammatory mediators secreted by adipocytes and macrophages play a major role in inhibiting the synthesis of the major extracellular matrix (ECM) components and exacerbating local inflammation during the initiation and perpetuation of the OA process [7, 8]. Obesity-related hyperlipemia is characterized by low systemic levels of high-density lipoprotein (HDL) and high levels of total cholesterol (TC), triglyceride (TG), and oxidized low-density lipoprotein (ox-LDL) [9]. Previous studies have pointed out that high serum TC and TG levels and lack of HDL have been linked to progression of cartilage loss in knee OA [10, 11]. In particular, lack of HDL predisposes mice to osteoarthritis following long-term exposure to high-fat diet (HFD) [12]. Furthermore, high systemic ox-LDL can promote VEGF release to induce the secretion of cartilage-destructive factors, such as catabolic enzymes MMP-1, MMP-3, and MMP-13 and proinflammatory mediators IL-1*β*, IL-6, and TNF-*α* [13–15]. In return, these proinflammatory mediators induce damage to cartilage, synovium, and subchondral bone by upregulating catabolic enzymes to degrade ECM [8].

Furthermore, new evidence suggests that the gut microbiota, through activating innate immune responses that result in systemic inflammation, represent a possible mechanistic link to metabolically induced OA [16]. It is currently established that activation of inflammation in obesity is also caused by shifts in the gut microbiota [17, 18]. Notably, HFD promotes disorder of the gut microbiota and enhances translocation of the bacterial membrane component lipopolysaccharide (LPS) into the bloodstream to conduct systemic and local inflammation [19]. Generally, this evidence emphasizes the pivotal roles for hyperlipemia and the gut microbiota in obesity-induced osteoarthritis. Accordingly, measures to reduce obesity and its related factors are regarded as effective strategies for inhibiting OA progression.

Acupuncture, a method of Chinese medicine, has been shown to balance pro- and anti-inflammatory cytokines, increase the release of neuropeptides and opioids, and regulate vasodilatation, via insertion of thin needles into specific points on the body [20]. Acupuncture may be a safe alternative to current pharmacological therapies for patients with osteoarthritis of the knee [21], but it is necessary to emphasize the specific acupuncture protocol of treatment best suited for OA in the future research, including duration and frequency of treatments, specific optimal acupoints for OA, and evaluation of acupuncture as adjuvant or as alternative treatment. Liangqiu (ST34), Dubi (ST35), and Xuehai (SP10), known as “knee three needling,” are the most frequently used acupoints in traditional acupuncture therapy of OA [22], and many acupuncture protocols with specific optimal acupoints based on “knee three needling,” such as supplementing Zusanli (ST36) and/or Yanglingquan (GB34), have been derived from clinical experiences. ST36 and GB34 acupoints have been tried for anti-inflammatory and analgesia effects of acupuncture stimulation [23–25], and the anti-inflammatory effect of the ST36 acupoint has been employed to treat obesity-related inflammatory diseases [26, 27]. However, the potential protective effects of traditional acupuncture protocols with ST36 and GB34 acupoints on obesity-induced OA remain unknown.

The aim of the present study was to investigate the effects of different acupuncture patterns on OA pathogenesis in HFD-induced obese rats. To address this objective, we employed an HFD-induced obesity model of knee OA and examined the impact of three electroacupuncture patterns ST36, GB34, and ST36+GB34 on OA pathogenesis of HFD-induced obese rats based on the traditional OA protocol.

## 2. Method

### 2.1. Animals and Diets

Thirty 8-week-old male SD rats, housed individually on a 12 h dark/light cycle, were purchased from a specific pathogen-free facility (Chengdu Dossy Experimental Animals Co., Ltd., Chengdu, China) and maintained at Southwest Medical University with standard monitoring thereafter. Animals were allocated to the HFD-induced obesity group (diet-induced obesity (DIO): 40% of total energy from fat, 45% of total energy from sucrose, Diet #102412, Dyets, Inc.) or the standard control chow diet group (12% fat, 3.7% sucrose, Lab Diet 5001) for a 12-week *ad libitum* feeding intervention. The HFD consisted of the following (g/100 g): casein (20.0), sucrose (49.9), soybean oil (10.0), lard (10.0), Alphacel (5.0), AIN-93M mineral mix (3.5), AIN-93 vitamin mix (1.0), DL-methionine (0.3), and choline bitartrate (0.25). The energy densities of the HFD and chow diets were 4.60 kcal/g and 3.34 kcal/g, respectively. All rats were weighed with an electronic scale per two weeks.

### 2.2. Electroacupuncture Manipulation

After a 12-week obesity induction period, DIO animals, whose body weight was overweight (more than 30% of the average body weight of control rats), were randomly divided into four groups (six per group): (i) diet-induced obese knee osteoarthritis models (DIO-KOA), (ii) diet-induced obesity following electroacupuncture on the ST36 acupoint (DIO-ST36), (iii) diet-induced obesity following electroacupuncture on the GB34 acupoint (DIO-GB34), (iv) diet-induced obesity following electroacupuncture on the ST36 and GB34 acupoints (DIO-ST36+GB34).

All the animals of DIO-ST36, DIO-GB34, and DIO-ST36+GB34 groups were all given electroacupuncture stimulation with the “knee three needling” acupoints of ST34 (Liangqiu, 2 mm deep), ST35 (Dubi, 2 mm deep), and SP10 (Xuehai, 3 mm deep). Differently, the animals in the DIO-ST36 group were inserted with acupuncture needles on ST36 (Zusanli, 5 mm deep), the animals in the DIO-GB34 group were inserted with acupuncture needles on GB34 (Yanglingquan, 4 mm deep), and the animals in the DIO-ST36+GB34 group were simultaneously inserted with acupuncture needles on ST36 and GB34. During acupuncture, animals were awake and immobilized using special cages to minimize stress. The disposable sterile acupuncture needles (0.3 mm × 13 mm) (Huatuo Medical Instruments Company, Suzhou, China) were inserted into those acupoints at a depth of 3 mm and connected to a HANS-200 electric stimulator (Han Shi, Nanjing, China) with an intensity of 2-3 mA and a frequency of 30 Hz for 10 min. Electroacupuncture manipulation was performed once daily for two weeks, alternately at the left and right sides of these points. Animals of the control and DIO-KOA groups were fixed for 10 min in the same way without electroacupuncture.

### 2.3. Measurement of Serum Total Cholesterol, Triglyceride, High-Density Lipoprotein, and Low-Density Lipoprotein

All rats were sacrificed after 2-week electroacupuncture treatment. Blood samples were collected from the abdominal aorta and were centrifuged at 1000 × g for 15 min and stored at -80°C. Serum total cholesterol (TC), triglycerides (TG), high-density lipoprotein (HLD), and low-density lipoprotein (LDL) were determined using commercial kits (Bio-Technology and Science Inc., Beijing, China). All experimental protocols were performed according to the manufacturer's instructions.

### 2.4. Assessment of OA

Joints were harvested by cutting the femur and tibia/fibula 2 cm above and below the joint line. Whole left knee joints were removed by dissection, were fixed in 4% paraformaldehyde for 24 h, and were decalcified in 10% EDTA-Na_2_ for two months at room temperature. After the joints underwent dehydration and paraffin embedding, serial sagittal sections (5 *μ*m) were cut from the whole medial compartment of the joint. The sections were stained sequentially with haematoxylin, fast green, and safranin-O stains (Fisher Scientific, MA, USA) and were examined microscopically. Sections were examined and scored for OA degeneration using a modified Mankin scoring system to describe the volumetric damage in each joint [28]. Five areas were evaluated: the medial and lateral tibial plateau, the medial and lateral femoral condyle, and the patella. The grading system included four categories: cartilage structure (six points), cartilage cells (three points), staining (four points), and tidemark integrity (two points). A maximum score of 14 points was possible, while normal cartilage received a score of zero points.

### 2.5. Immunohistochemistry

Briefly, tissue sections were incubated overnight at 4°C with an anti-rat MMP-1 antibody and anti-rat MMP-13 antibody, respectively (1 : 100; Boster Biotechnology, Wuhan, Hubei, China) and then with an appropriate secondary antibody (Boster Biotechnology, Wuhan, China) at 37°C. After 30 min, bound antibodies were visualized using peroxidase-conjugated avidin and diaminobenzidine according to the manufacturer's instructions (Boster Biotechnology, Wuhan, China). Cartilage areas were selected, and staining signals were quantified and averaged using the ImageJ program (Media Cybernetics, Carlsbad, CA, USA).

### 2.6. Measurement of Cytokine, Growth Factor, Adipokine, and LPS in Serum and Articular Synovial Fluid

Synovial fluid was collected shortly after sacrifice using the Whatman chromatography paper method [29]. Synovial fluid from the left and right limbs of each animal was pooled for quantification. Serum and synovial fluid cytokines (MIP-1*α*, MIP-2, IP-10, IL-1*α*, TNF-*α*, and MCP-1), VEGF, and leptin were quantified by ELISA (Cusabio, Wuhan, Hubei, China). LPS level in serum and synovial fluid was evaluated using the EndoZyme Recombinant Factor C Assay (Hyglos GmbH, Germany). All experimental protocols were performed according to the manufacturers' instructions.

### 2.7. Western Blotting

Arthrodial cartilage was used for protein extraction. After arthrodial cartilage collection, the arthrodial cartilage of rats was crushed using a mortar and pestle in liquid nitrogen and then lysed with RIPA buffer in the presence of 1% protease inhibitor cocktail (Roche, Basel, Switzerland). Protein from cell cultures was also extracted using RIPA buffer. The supernatant was collected after centrifugation at 12,000 g and 4°C for 30 min. Protein concentration was quantified with the BCA Protein Assay Kit (Generay, Shanghai, China). After being denatured in boiling for 5 min in SDS sample buffer, 40 *μ*g of total protein was separated by 6%-15% SDS-PAGE, blotted onto PVDF membranes, and then probed with the following antibodies (Cell Signaling Technology, Danvers, MA, USA): monoclonal anti-TLR4 antibody, monoclonal anti-NF-*κ*B p65 antibody, monoclonal anti-NF-*κ*B phosphorylated p-65 (P-p65) antibody, and mouse anti-*β*-action antibody, conjugated to horseradish peroxidase, which were used as secondary antibodies. Protein bands were visualized by incubation with BeyoECL Plus (P0018, Beyotime, China) for 1 min and imaged by a Gel Image System (Tanon, 5200, China). Densitometry was performed by using the enhanced chemiluminescence (ECL) detection system (Thermo Scientific, Rockford, IL, USA).

### 2.8. 16S rRNA Bacterial Sequencing

Fecal pellets from three rats per group were freshly harvested from rats after sacrifice and immediately frozen at -80°C. DNA was extracted using the ZR Fecal DNA Extraction Kit (Zymo Research, CA, USA) as directed by the manufacturer. 16S ribosomal DNA (rDNA) was amplified with Phusion High-Fidelity Polymerase (Thermo Fisher Scientific) and dual-indexed primers specific to the V3-V4 hypervariable regions (319F: 5′ ACTCCTACGGGAGGCAGCAG 3′, 806R: 3′ ACTCCTACGGGAGGCAGCAG 5′). Amplicons were pooled and paired-end sequenced on Illumina MiSeq (Illumina) in Shanghai Personal Biotechnology Co., Ltd. (Shanghai, China). The Quantitative Insights Into Microbial Ecology (QIIME, v1.8.0) pipeline was employed to process the sequencing data, as previously described. Sequence processing and microbial composition analysis were performed with the Quantitative Insights Into Microbial Ecology (QIIME) software package version 1.9.1. After quality filters, the remaining high-quality sequences were clustered into operational taxonomic units (OTUs) at 97% sequence using the reference-based USEARCH (version 5.2) pipeline in QIIME, using the May 2013 release of the Greengenes 99% OTU database as a closed reference. The raw data and sequencing sample information have been submitted to the SILVA database to be classified.

### 2.9. Statistical Analysis

Data were presented as the mean ± standard deviation (SD). The SPSS software package 19.0 (SPSS Inc., Chicago, IL, United States) was used for the statistical calculations in this study. After verification of a normal or nonnormal distribution, two-tailed Student's *t*-test and *post hoc* ANOVA were performed to analyze the variables of normal distribution. When data was not normally distributed, it was log-transformed. A *p* value less than 0.05 was considered significant.

## 3. Results

### 3.1. Electroacupuncture Attenuated Lipid Metabolic Disorders Induced by High-Fat Diet

**Figure 1:**
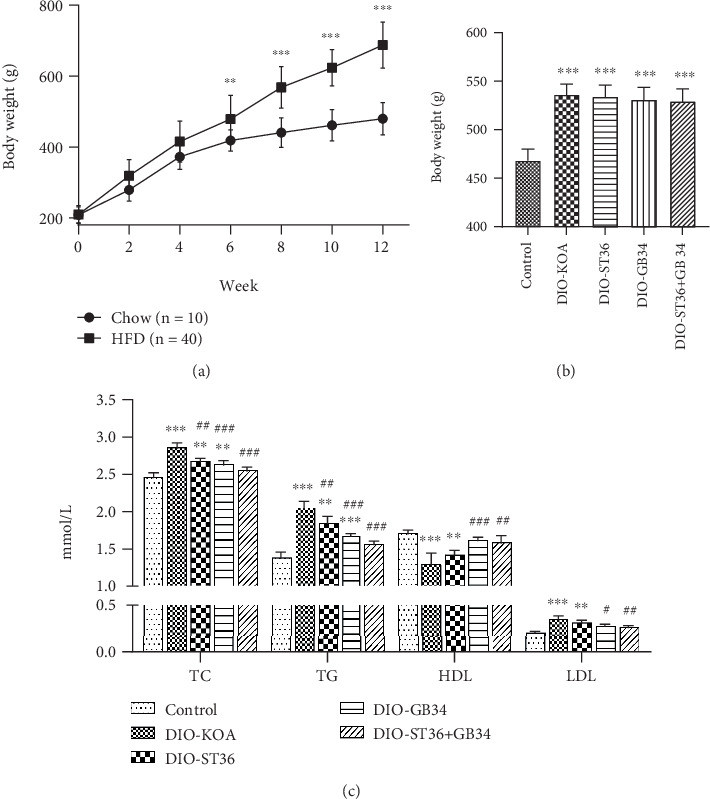
Effect of electroacupuncture on body weight and the lipid metabolic profile of obese rats. (a) After 12-week feeding, body weight of rats fed with HFD gradually increased to be overweight than that of chow-fed rats. Data were expressed as mean ± SD (6 rats in control and 24 rats in HFD); ^∗^*p* < 0.05, ^∗∗^*p* < 0.01, and ^∗∗∗^*p* < 0.001 analyzed by two-tailed Student's *t*-test. (b) Body weights were measured after 2-week electroacupuncture treatments. (c) Serum levels of total cholesterol (TC), triglyceride (TG), high-density lipoprotein (HDL), and low-density lipoprotein (LDL) were measured after 2-week electroacupuncture treatments. All data are expressed as the mean ± SD (*n* = 6 per group). *Post hoc* ANOVA was used to test for statistical significance. ^∗^*p* < 0.05, ^∗∗^*p* < 0.01, and ^∗∗∗^*p* < 0.001 versus the control group; ^#^*p* < 0.05, ^##^*p* < 0.01, and ^###^*p* < 0.001 versus the DIO-KOA group.

To model the pathological process of the knee osteoarthritis (KOA) of obesity in humans, rats were fed with HFD for 12 weeks to induce obesity, and the body weight of HFD-fed rats was overweight compared with chow-fed rats (Figure 1(a)). Thereafter, those diet-induced obesity rats were divided into four groups to receive 2-week electroacupuncture treatments.

Interestingly, we found that electroacupuncture treatments of ST36, GB34, and ST36+GB34 could not reduce the body weight of obesity rats (Figure 1(b)) and the amount of food intake (data not shown) but attenuated lipid metabolic disorders induced by HFD. As shown in Figure 1(c), obesity rats of the DIO-KOA group showed a disturbed lipid metabolism characterized by low serum levels of HDL and high levels of TC, TG, and LDL. Serum levels of TC and TG were both significantly decreased by three electroacupuncture treatments, but the inhibition of ST36+GB34 on TC and TG was better than that of ST36 or GB34. Furthermore, GB34 and ST36+GB34 also increased HDL level and decreased LDL level compared with the DIO-KOA group.

### 3.2. Electroacupuncture Prevents Cartilage Loss in the OA of Diet-Induced Obesity Rats

**Figure 2:**
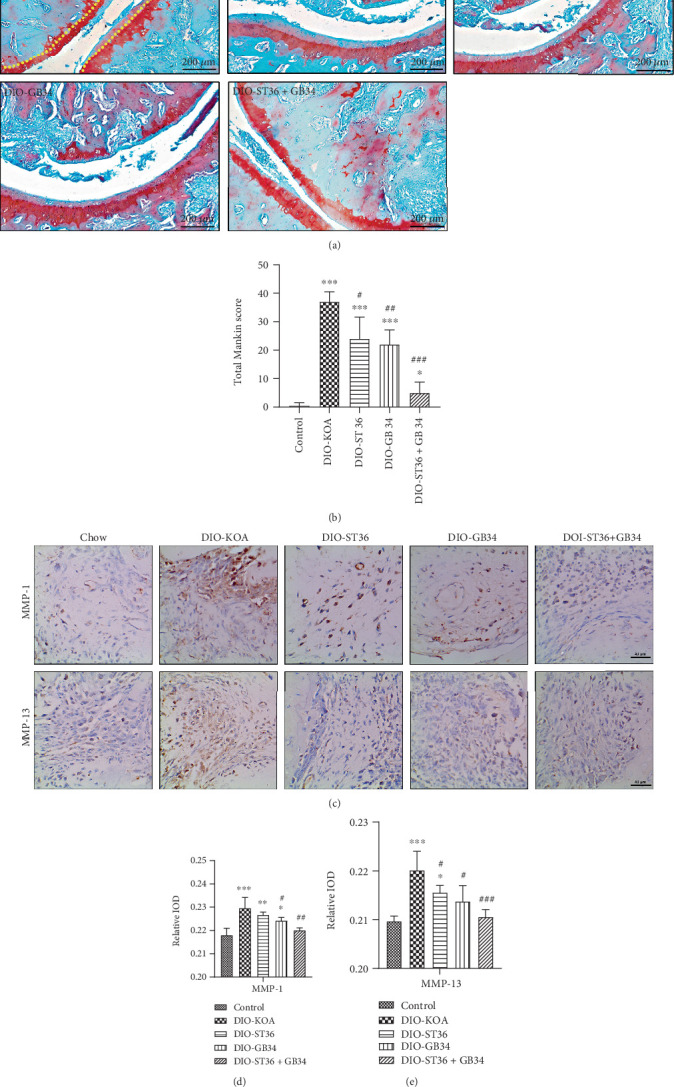
Electroacupuncture prevents cartilage loss in the OA of diet-induced obesity rats. (a) Representative safranin-O/fast green-stained sections. Scale bar = 200 *μ*m. (b) Quantification of modified total Mankin scores. (c) Representative sections of knee joints that were stained for MMP-1 and MMP-13. Scale bar = 40 *μ*m. (d) The integral absorbency of MMP-1 in cartilage areas. (e) The integral absorbency of MMP-13 in cartilage areas. All data are expressed as the mean ± SD (*n* = 6 per group). *Post hoc* ANOVA was used to test for statistical significance. ^∗^*p* < 0.05, ^∗∗^*p* < 0.01, and ^∗∗∗^*p* < 0.001 versus the control group; ^#^*p* < 0.05, ^##^*p* < 0.01, and ^###^*p* < 0.001 versus the DIO-KOA group.

Obese rats of the DIO-KOA group without any electroacupuncture treatment showed typical characteristics of OA, including loss of tide lines, a reduction in cartilage thickness, and the presence of articular cartilage lesions in the knee joint (Figure 2(a)). In contrast, electroacupuncture treatments of ST36, GB34, and ST36+GB34 resulted in some improvement in matrix arrangement, tide line maintenance, and cartilage lesion inhibition (Figure 2(a)). In the total Mankin score summed with the medial and lateral tibial plateau and the medial and lateral femoral ankle score, obesity rats demonstrated significantly higher total Mankin knee joint scores than the chow-fed control rats, but those rats treated with electroacupuncture showed a lower score compared with obesity rats (Figure 2(b)). Furthermore, the increased expressions of MMP-1 and MMP-13 in arthrodial cartilage of obese rats were decreased by the electroacupuncture treatments in the ST36, GB34, and ST36+GB34 groups, which proved that electroacupuncture prevented the degradation of the cartilage matrix induced by high-fat diet (Figures 2(c)–2(e)). Remarkably, ST36+GB34-treated obese rats were almost completely rescued from the deleterious effect of obesity, with more effectiveness in preventing cartilage loss and reducing the total Mankin score and the expression of MMP-1 and MMP-13.

### 3.3. Electroacupuncture Attenuated Systemic and Knee Inflammation of Obese Rats

**Figure 3:**
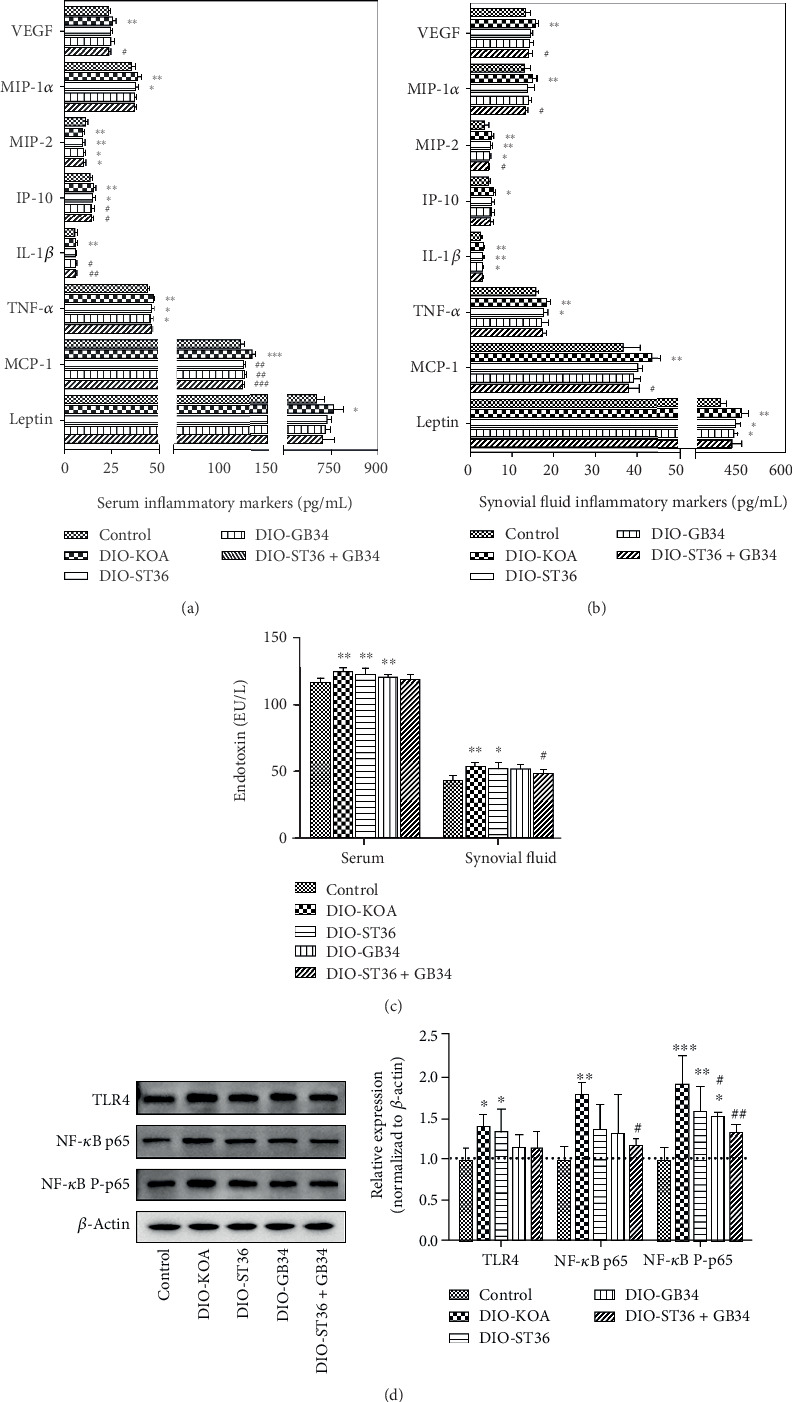
Electroacupuncture attenuated systemic and knee inflammation of obese rats. (a) Serum inflammatory markers. (b) Synovial fluid inflammatory markers. (c) Serum and synovial fluid LPS level. (d) Western blot analysis of TLR4, NF-*κ*B p65, and phosphorylated p65 (P-p65) expression in arthrodial cartilage. All data are expressed as the mean ± SD (*n* = 6 per group). *Post hoc* ANOVA was used to test for statistical significance. ^∗^*p* < 0.05, ^∗∗^*p* < 0.01, and ^∗∗∗^*p* < 0.001 versus the control group; ^#^*p* < 0.05, ^##^*p* < 0.01, and ^###^*p* < 0.001 versus the DIO-KOA group.

In serum and synovial fluid, eight obesity-induced inflammatory cytokines (VEGF, MIP-1*α*, MIP-2, IP-10, IL-1*α*, TNF-*α*, MCP-1, and leptin) and LPS were increased in the DIO-KOA group compared with the control group (Figure 3). Electroacupuncture with ST36 or GB34 could reduce those cytokine levels, but there was no significant difference in most inflammatory cytokines between the DIO-KOA group and ST36 or GB34 group (Figure 3(a)). However, electroacupuncture with ST36+GB34 significantly decreased four inflammatory cytokines (VEGF, IP-10, IL-1*α*, and MCP-1) in serum (Figure 3(a)) as well as VEGF, MIP-1*α*, MIP-2, and MCP-1 in synovial fluid (Figure 3(b)). Furthermore, we found that ST36+GB34 decreased the LPS level especially in synovial fluid (Figure 3(c)). LPS plays an important role in inducing inflammatory response of obesity-related metabolic diseases by activating the TLR4/NF-*κ*B signaling pathway [30]. The expression levels of TLR4, p65, and the activated form of p65 (P-p65) in the arthrodial cartilage were significantly higher in the DIO-KOA group compared with the control group, which indicated that LPS participated in the knee inflammation of obese rats by activating the TLR4/NF-*κ*B signaling pathway. Moreover, we found that GB34 inhibited the expression of P-p65, while ST36+GB34 could inhibit the expression of p65 and P-p65.

### 3.4. Electroacupuncture Increased the Microbial Diversity and Altered the Structure of the Community of Feces in Obese Rats

**Figure 4:**
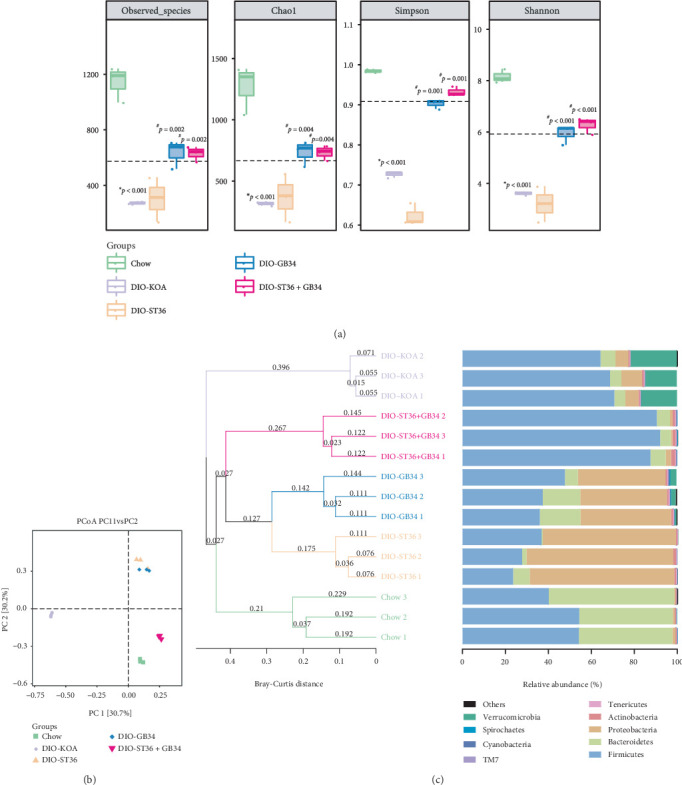
Electroacupuncture increased the microbial diversity and altered the structure of the community of feces in obese rats. (a) Richness and diversity of the fecal microbiota. All data are expressed as the mean ± SD (*n* = 3 per group). *Post hoc* ANOVA was used to test for statistical significance. ^∗^*p* < 0.05, ^∗∗^*p* < 0.01, and ^∗∗∗^*p* < 0.001 versus the control group; ^#^*p* < 0.05, ^##^*p* < 0.01, and ^###^*p* < 0.001 versus the DIO-KOA group. (b) Principal coordinate analysis (PCoA) of the Bray-Curtis distance. (c) Cluster analysis of the unweighted pair group method with arithmetic mean (UPGMA) based on the Bray-Curtis distance.

The gut microbiome is closely associated with lipid metabolism disorder and systemic inflammation of obese rats [31]; thus, fecal samples were collected to analyze the gut microbiome of obese rats after 2-week acupuncture treatments. Multiple alpha diversity metrics of richness and diversity revealed a lower microbial diversity in the DIO-KOA group compared with the control group, but the multiple alpha diversity metrics of observed species, Chao1, Simpson, and Shannon, were all increased by acupuncture treatments of GB34 and ST36+GB34 (Figure 4(a)). Bray-Curtis distance-based PCoA was employed to uncover the similarities and differences in the composition of the gut microbiota among control, DIO-KOA, and three acupuncture groups. The DIO-KOA group showed an obvious difference in clustering of the gut microbiota compared with the control group. Acupuncture groups of ST36, GB34, and ST36+GB34 exhibited a movement in the first principal component (PC1) towards the direction of the control group, but the ST36 group and GB34 group both showed opposite directions of movement in the PC2 axis relative to the ST36+GB34 group; thus, ST36+GB34 shortened the distance with the control group (Figure 4(b)). Hierarchical clustering analysis revealed that the microbial communities in three acupuncture-treated groups showed more similarities to those in the control group (Figure 4(c)). Furthermore, the ST36+GB34 group also showed a distinguishing gut microbiota with the ST36 group or GB34 group (Figures 4(b) and 4(c)). Overall, three acupuncture treatments significantly reverted the obesity-induced variations along PC1 but separately contributed to a diverse shift in the structure along PC2.

### 3.5. Electroacupuncture-Associated Alterations in the Fecal Microbiota

**Figure 5:**
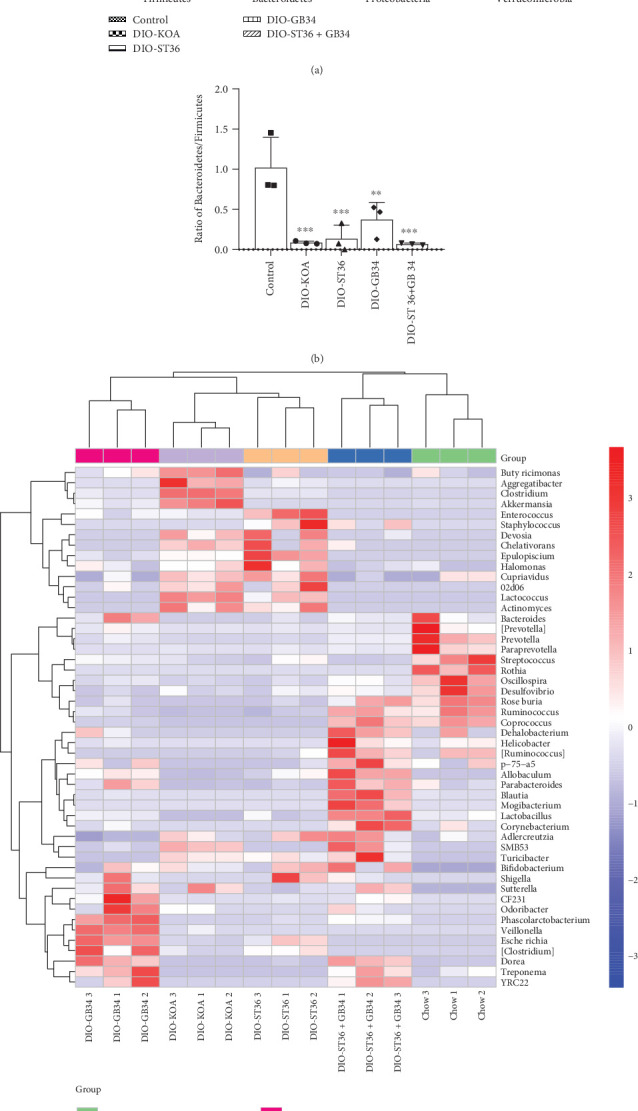
Acupuncture-associated alterations in the fecal microbiota. (a) Comparison of the relative abundance of *Firmicutes*, *Bacteroidetes*, *Proteobacteria*, and *Verrucomicrobia* phyla within groups. (b) The ratio of *Bacteroidetes*/*Firmicutes* at the phylum level. All data are expressed as the mean ± SD (*n* = 3 per group). *Post hoc* ANOVA was used to test for statistical significance. ^∗^*p* < 0.05, ^∗∗^*p* < 0.01, and ^∗∗∗^*p* < 0.001 versus the control group; ^#^*p* < 0.05, ^##^*p* < 0.01, and ^###^*p* < 0.001 versus the DIO-KOA group. (c) The heat map of the top 50 abundant genera. Double hierarchical dendrogram shows the bacterial distribution.

Interestingly, there were similar microbial structures between ST36 and GB34 groups, but the effect of the ST36+GB34 pattern on the composition of the gut microbiota was not the superimposed effect of ST36 and GB34. As shown in Figures 5(a) and 5(b), DIO-related changes in the fecal microbiota were characterized by higher relative abundance of *Firmicutes*, *Proteobacteria*, and *Verrucomicrobia*, as well as lower relative abundance of *Bacteroidetes* and the ratio of *Bacteroidetes*/*Firmicutes* at the phylum level. After 2-week electroacupuncture treatments, ST36 and GB34 both significantly decreased the relative abundance of *Firmicutes* and increased the relative abundance of *Proteobacteria*. However, ST36+GB34 significantly increased the relative abundance of *Firmicutes* and decreased the relative abundance of *Proteobacteria*. Uniformly, the relative abundance of *Verrucomicrobia* was decreased by ST36, GB34, and ST36+GB34. Furthermore, the three electroacupuncture patterns could not increase the ratio of *Bacteroidetes*/*Firmicutes*.

**Figure 6:**
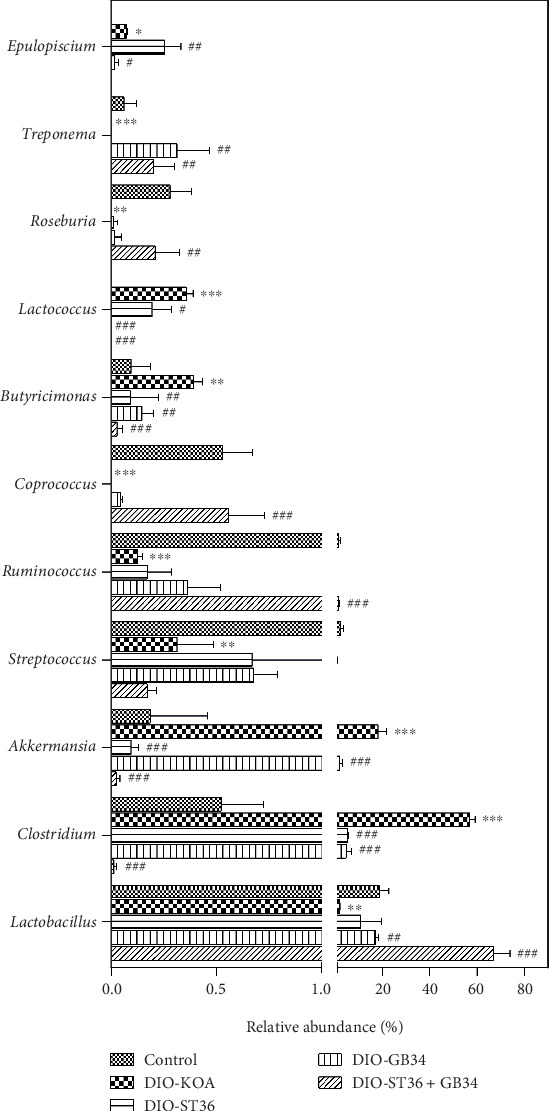
Acupuncture-associated alterations at the genus level. All data are expressed as the mean ± SD (*n* = 3 per group). Post hoc ANOVA was used to test for statistical significance. ^∗^*p* < 0.05, ^∗∗^*p* < 0.01, and ^∗∗∗^*p* < 0.001 versus the control group; ^#^*p* < 0.05, ^##^*p* < 0.01, and ^###^*p* < 0.001 versus the DIO-KOA group.

Consistent with beta diversity, clustering analysis of the top 50 genera highlighted differences in their distributions due to electroacupuncture treatments (Figure 5(c)). As shown in Figure 6, compared with the control group, obese rats in the DIO-KOA group showed a higher level in the relative abundances of *Clostridium*, *Akkermansia*, *Butyricimonas*, *Lactococcus*, and *Epulopiscium* and a lower level in the relative abundances of *Lactobacillus*, *Streptococcus*, *Ruminococcus*, *Coprococcus*, *Roseburia*, and *Treponema*. The three electroacupuncture patterns of ST36, GB34, and ST36+GB34 all promoted the recovery of relative abundance of *Clostridium*, *Akkermansia*, *Butyricimonas*, and *Lactococcus* with different efficiencies. Furthermore, the relative abundances of *Lactobacillus* and *Treponema* were increased by GB34 and ST36+GB34, and the relative abundances of *Coprococcus* and *Roseburia* were increased by ST36+GB34. However, the relative abundance of *Epulopiscium* was increased by ST36 but decreased by GB34, while there is no difference in ST36+GB34.

## 4. Discussion

Obesity-related metabolic syndrome and corresponding chronic low-grade systemic inflammation are associated with the onset and progression of osteoarthritis (OA) [32]. In this study, we compared the effects of different electroacupuncture patterns on inflammation, gut microbiota, and knee joint damage in the context of HFD diet-induced obesity. As a result, we found that the traditional OA acupuncture protocol with ST36 and GB34 acupoints reduced obesity-induced knee joint damage by regulating lipid metabolism and alleviating chronic low-grade systemic inflammation induced by gut microbiota disorder.

Obesity is thought to be a major risk factor for the development of OA, not only because excessive weight leads to wear and tear of the joint but also because inflammation and dyslipidemia play a pivotal role in obesity-induced OA [5]. Obesity precedes the development of OA and thus is deemed to be causally implicated in the degenerative changes that underlie OA [33]. In the present study, rats fed with HFD in the absence of trauma exhibited OA-like changes in the knee joints after a standard 12-week obesity induction period, which was concordant with previous studies that HFD-induced obesity increased the incidence and degeneration of OA [28, 34, 35]. Indeed, high loading results in cartilage degradation and subchondral bone changes, indicating that weight is important in the initiation and progression of OA. However, the prevalence of hypertrophied adipose tissue results in macrophage infiltration with subsequent inflamed adipose tissue [26]. Many of the proinflammatory mediators are secreted by the adipocytes, whereas others are predominantly derived from adipose tissue-infiltrated macrophages. The proinflammatory mediators secreted by the inflamed adipose tissue increased the susceptibility to OA, which can induce damage to the cartilage, synovium, and subchondral bone by upregulating ECM-degrading enzymes and inflammatory mediators [7, 8].

Concordant with previous reports [28, 35], increases in the magnitude of local inflammatory markers in synovial fluid and serum were observed in obese rats, including VEGF, MIP-1*α*, MIP-2, IP-10, IL-1*α*, TNF-*α*, MCP-1, and leptin. The role of these inflammatory cytokines in activating and maintaining obesity-induced systems and local inflammatory has been demonstrated [5, 8]. ST36 and GB34 acupoints have been tried for anti-inflammatory and analgesia effects of electroacupuncture stimulation [23–25]. It is reported that alone acupuncture on the ST36 acupoint can decrease the leukocyte infiltration to adipose tissue and attenuates inflammatory response by reducing serum levels of TNF-*α*, IL-6, and IL-1 in HFD-induced obesity rats [26]. For unilateral knee ligament transaction inducing knee degeneration, the traditional OA acupuncture protocol (ST34, ST35, and SP10) with ST36 and GB34 has potential beneficial effects on relieving pain and inhibiting the secretion of inflammatory cytokines and the degradation of the cartilage matrix in OA rat models [36]. Nevertheless, the three electroacupuncture patterns of ST36, GB34, and ST36+GB34 all prevented obesity-induced cartilage matrix degradation and MMP expression, but the three electroacupuncture patterns had different regulatory effects on obesity-induced systemic and local inflammation cytokines. The traditional OA acupuncture protocol with ST36 only significantly decreased the MCP-1 level in serum, while the protocol with GB34 significantly decreased the level of IP-10, IL-1*α*, and MCP-1 in serum. However, simultaneous acupuncture on ST36 and GB34 not only mitigated IP-10, IL-1*α*, and MCP-1 in serum but also reduced VEGF, MIP-1*α*, MIP-2, MCP-1, and LPS in synovial fluid. These results suggested that acupuncture on different acupoints had different anti-inflammatory effects, while simultaneous acupuncture on ST36 and GB34 was better for inhibiting joint inflammation.

Obesity-associated hyperlipemia also can contribute to joint inflammation and cartilage destruction of OA. High serum TC and TG levels and parafunctional HDL have been associated with the occurrence of bone marrow lesions [12], which is a source of pain in OA and may lead to progression of cartilage loss in knee OA [37]. After 12 weeks of being administered HFD, the obese rats exhibited low systemic levels of HDL and high levels of TC, TG, and LDL, which were in accordance with the characteristics of disturbed lipid metabolism in obese rats [9]. Electroacupuncture on ST36 or GB34 has been reported to inhibit lipogenesis by decreasing the level of LDL, TC, and TG in serum of diet-induced obesity or hyperlipidemia rats [27, 38]. Thereto, the lipid-lowering effect of ST36 and GB34 has been linked to the attenuation of the expression of sterol regulatory element-binding protein in the liver of hyperlipidemic rats [39] and the regulation of the neuroendocrine pathways to promote lipid metabolism of obesity rats [27]. Interestingly, the traditional OA acupuncture protocol with ST36, GB34, and ST36+GB34 could not reduce the body weight of obesity rats, but the protocol with ST36 and GB34 attenuated lipid metabolic disorders induced by HFD. The serum levels of TC and TG were both decreased by the three electroacupuncture protocols. Furthermore, GB34 and ST36+GB34 also increased HDL level and decreased LDL level of obese rats. Normally, HDL scavenges cholesterol and other lipids from the bloodstream and transports them to the liver, thereby maintaining lipid homeostasis [40]. In contrast, high LDL promotes cholesterol and lipid transfer outside the liver and results in the deposition of cholesterol and lipid under knee cartilage [37]. High cholesterol not only promotes the activation of synovial macrophages but also promotes the uptake of ox-LDL in synovial macrophages [41], which can stimulate VEGF release from chondrocytes to induce joint inflammation and cartilage destruction through increasing the expression of catabolic enzymes MMP-1 and MMP-13 and the proinflammatory mediators IL-1*β*, IL-6, and TNF-*α* [14]. Therefore, decreasing the deposition of cholesterol and lipid under knee cartilage to relieve synovial inflammation by regulating lipid metabolism may be an important mechanism for acupuncture on ST36 and GB34 to promote OA recovery.

It is currently recognized that the gut microbiome can have a profound influence on systemic inflammation and chronic disease. Roles of key microbial species in inducing inflammation or protecting from it have already been established in the obesity-induced OA [28, 42]. Consistent with previous work, induction of obesity via consumption of HFD led to a proinflammatory shift in the gut microbiome characterized by a decrease in Bacteroidetes abundance and the ratio of Bacteroidetes/Firmicutes. Key changes included ablation of beneficial microbes from the genera *Lactobacillus*, *Streptococcus*, and *Ruminococcus* coupled with increased abundance of *Clostridium* and *Akkermansia*, which are associated with obesity-induced inflammation [43, 44]. Importantly, it has been confirmed in an obesity-induced OA animal model that this proinflammatory microbial shift coincided with an increase in key proinflammatory cytokines in the circulation, increased macrophage presence in the knee capsule, and upregulation of the monocyte chemokine MCP-1 in synovial tissue [42]. In this study, we found that the three electroacupuncture patterns of ST36, GB34, and ST36+GB34 all promoted the recovery of relative abundance of *Clostridium*, *Akkermansia*, *Butyricimonas*, and *Lactococcus* with different efficiencies. Therefore, the three electroacupuncture patterns of ST36, GB34, and ST36+GB34 could reduce the risk of obesity-induced OA by promoting the abundance of beneficial microbes to relieve systemic inflammation of obesity rats.

The gut microbiota is considered to be a key environmental factor determining the tendency to obesity and lipid metabolism disorders [45]. It plays an important role in balancing cholesterol levels via producing cholesterol oxidase to accelerate cholesterol degradation [46]. Furthermore, it can also regulate cholesterol metabolism by producing short-chain fatty acids to inhibit cholesterol synthesis in the liver or redistribute cholesterol from plasma to the liver [47]. In particular, the genus *Lactobacillus*, as a dominant microbiota in the gut, plays a particularly important role in clearing cholesterol [46]. Interestingly, we found that the relative abundance of the beneficial genus *Lactobacillus* was significantly increased by GB34 and ST36+GB34, which was consistent with the effect of GB34 and ST36+GB34 on LDL and HDL in serum. It is reported that *Lactobacillus* can increase HDL and decrease LDL to clear cholesterol in serum [46]. These results suggested that GB34 might play a role in regulating the levels of LDL and HDL by increasing *Lactobacillus*.

As a product of the gut microbiota, the increased LPS in serum and synovial fluid of HFD-induced obesity rats also provided a link between the associations measured between gut microbes, chronic inflammation, and increased joint damage, because LPS participated in activating macrophages in arthrodial cartilage of obesity rats, through stimulating toll-like receptor (TLR) 2/4 and activating the NF-*κ*B signaling pathway, subsequently promoting the secretion of proinflammatory mediators such as TNF-*α* [48]. Although alone the acupuncture protocol with ST36 or GB34 could not significantly inhibit LPS-mediated NF-*κ*B activation, the electroacupuncture protocol with ST36+GB34 decreased the LPS level in synovial fluid and inhibited the phosphorylation of NF-*κ*B p65 in arthrodial cartilage of obesity rats. This result indicated that the OA acupuncture protocol with ST36+GB34 might be more influential to inhibit LPS-mediated joint inflammation in obesity rats.

Regarding the antiobesity effects of the electroacupuncture treatment on the gut microbiota structure, it is not clear whether these variations in the gut microbiota are a causal risk factor for obesity-related diseases or only reflect electroacupuncture treatment. Most of the researchers believed that the relationship between the gut microbiota and electroacupuncture depends on the brain-gut axis theory [49, 50]. The gut microbiota may directly or indirectly target the brain through vagal stimulation or the immune-neuroendocrine mechanism, thereby regulating food intake and gastrointestinal motility [50, 51], which is essential for the development of obesity. Recent studies have indicated that electro- and manual acupuncture can activate and release brain-gut peptide and appetite-related hormones in the hypothalamus, followed by mediating food intake, gastrointestinal motility, and gut microbiota [52–54]. Collectively, these findings suggest a potential role of the brain-gut axis in controlling appetite and regulating lipid and glucose metabolism during electroacupuncture treatment. Further studies are warranted to reveal the exact mechanisms underlying the effects of acupuncture on the modulation of the gut microbiota, thereby participating in regulating lipid metabolism and inflammation response in obesity-related disease.

In conclusion, our findings demonstrated that HFD fed to rats increased the risk of knee OA, and the electroacupuncture protocol with ST36 and GB34 could prevent obese rats from the loss of articular cartilage, through regulating the lipid metabolism and gut microbiota to inhibit joint inflammation. The possible mechanisms whereby acupuncture with different acupoints alters gut microbiota diversity and lipid metabolism require further investigation, but our results support the use of electroacupuncture in controlling or treating obesity- and hyperlipemia-related OA, thereby highlighting the potential preventive or therapeutic value of acupuncture for obesity-associated OA.

## Data Availability

The initial data used to support the findings of this study are available from the corresponding author upon request.
